# School outbreak of hand, foot and mouth disease in Balungao, Pangasinan Province, Philippines, October 2022

**DOI:** 10.5365/wpsar.2023.14.2.1001

**Published:** 2023-04-28

**Authors:** Emeryn C Victori, Ray Justin C Ventura, Mariz Zheila C Blanco, Rosario P Pamintuan, Rio L Magpantay, Karen B Lonogan

**Affiliations:** a Field Epidemiology Training Program – Intermediate Course, San Fernando City, La Union, Philippines.; b Center for Health and Development 1, Department of Health, San Fernando City, La Union, Philippines.

## Abstract

**Objective:**

On 24 September 2022, the Regional Public Health Unit in Ilocos received a report of a cluster of suspected hand, foot and mouth disease (HFMD) in one school in Balungao, Pangasinan Province, the Philippines. On 4 October 2022, the public health unit sent a team from the Field Epidemiology Training Program – Intermediate Course to conduct an outbreak investigation.

**Methods:**

Active case-finding was conducted at the school. A suspected case was defined as any student or staff member with mouth ulcers and papulovesicular or maculopapular rash on the palms, fingers, soles of the feet or buttocks occurring from 1 September to 5 October 2022. We interviewed school officials about possible sources of infection and students’ activities. We collected oropharyngeal swab samples for testing. Findings were used for descriptive analysis.

**Results:**

Nine suspected cases of HFMD were detected, with the highest number of cases (6, 67%) occurring in children in grade 1. The majority of cases (7, 78%) were 6 years old, and five cases (56%) were male. Seven (78%) of the cases had been exposed to a confirmed case of HFMD, as reported by their parents or guardians and teachers. Six cases (67%) were positive for coxsackievirus A16 and two (22%) for enterovirus.

**Discussion:**

The causative agents of this outbreak were coxsackievirus A16 and other enteroviruses. Direct contact with a confirmed case was the source of transmission, with a lack of physical distancing in classrooms likely contributing to transmission. We recommended that the local government implement measures to control the outbreak.

On 24 September 2022, the Regional Public Health Unit in Ilocos received a report of a cluster of suspected hand, foot and mouth disease (HFMD) cases in one school in Balungao, Pangasinan Province, the Philippines, from the Development Management Officer of the municipality. The outbreak was verified through the event-based surveillance and response system. On 4 October 2022, a team from the Field Epidemiology Training Program – Intermediate Course in Northern Luzon was dispatched to conduct an outbreak investigation.

HFMD is a common viral illness that usually affects infants and children younger than 5 years, although it can sometimes occur in older children and adults. Symptoms include low-grade fever, mouth sores and skin rashes. The rash is commonly found on the hands and feet, and sometimes on the genitals and buttocks. (*1*) A case is most contagious during the first week of the illness, but can be contagious for weeks after symptoms resolve. People without symptoms can still spread the virus. (*2*) HFMD is not transmitted to or from pets or other animals. (*2*)

Balungao municipality has an estimated population of 30 004, as per the 2020 census. (*3*) The school involved in the outbreak has 565 students enrolled from kindergarten to grade 6, ranging in age from 5 to 12 years.

## METHODS

Active case-finding was conducted at the school. A suspected case was defined as any student or staff member with mouth ulcer and papulovesicular or maculopapular rash on the palms, fingers, soles of the feet or buttocks occurring from 1 September to 5 October 2022. A confirmed case was a suspected case who tested positive for a human enterovirus that causes HFMD. Findings were used for descriptive analysis.

Face-to-face interviews were conducted with the parents or guardians of cases and teachers, using a standard questionnaire to collect information about demographic characteristics, clinical symptoms and exposure history. The medical records of cases who consulted with or were admitted to the local medical clinic or regional medical and trauma centre from 1 September to 5 October 2022 were also reviewed, as was 5-year HFMD surveillance data from the local health unit and the provincial epidemiology and surveillance unit of Pangasinan Province. From this, we developed a line list using Microsoft Excel that included the name, age, sex, address, grade level, date of onset and admission, signs and symptoms, possible source of infection and laboratory results of each case. The descriptive analysis included information about time (i.e. the scope of the study), place and person, with the frequencies and percentages of HFMD characteristics calculated using Microsoft Excel. An epidemic curve was created by date of onset to describe the epidemiological linkage of cases.

Interviews were also conducted using a guided questionnaire with the Municipal Health Officer, Disease Surveillance Officer, other health staff and school staff to determine possible sources of infection and to understand the activities and practices of students in the school and other relevant information. A site visit to school classrooms and grounds was conducted at the same time.

Oropharyngeal swab samples were collected and specimens placed in viral transport medium before being sent for testing to the Research Institute for Tropical Medicine in Alabang Muntinlupa City. Semi-nested polymerase chain reaction (PCR) was used for enterovirus detection, and an enterovirus multiplex reverse transcription–PCR was used to detect enterovirus 71, coxsackievirus A6 (CV-A6) and CV-A16.

A transmission pattern was observed and key areas contributing to the spread of the disease were identified.

## RESULTS

### Descriptive analysis

**Fig. 1:**
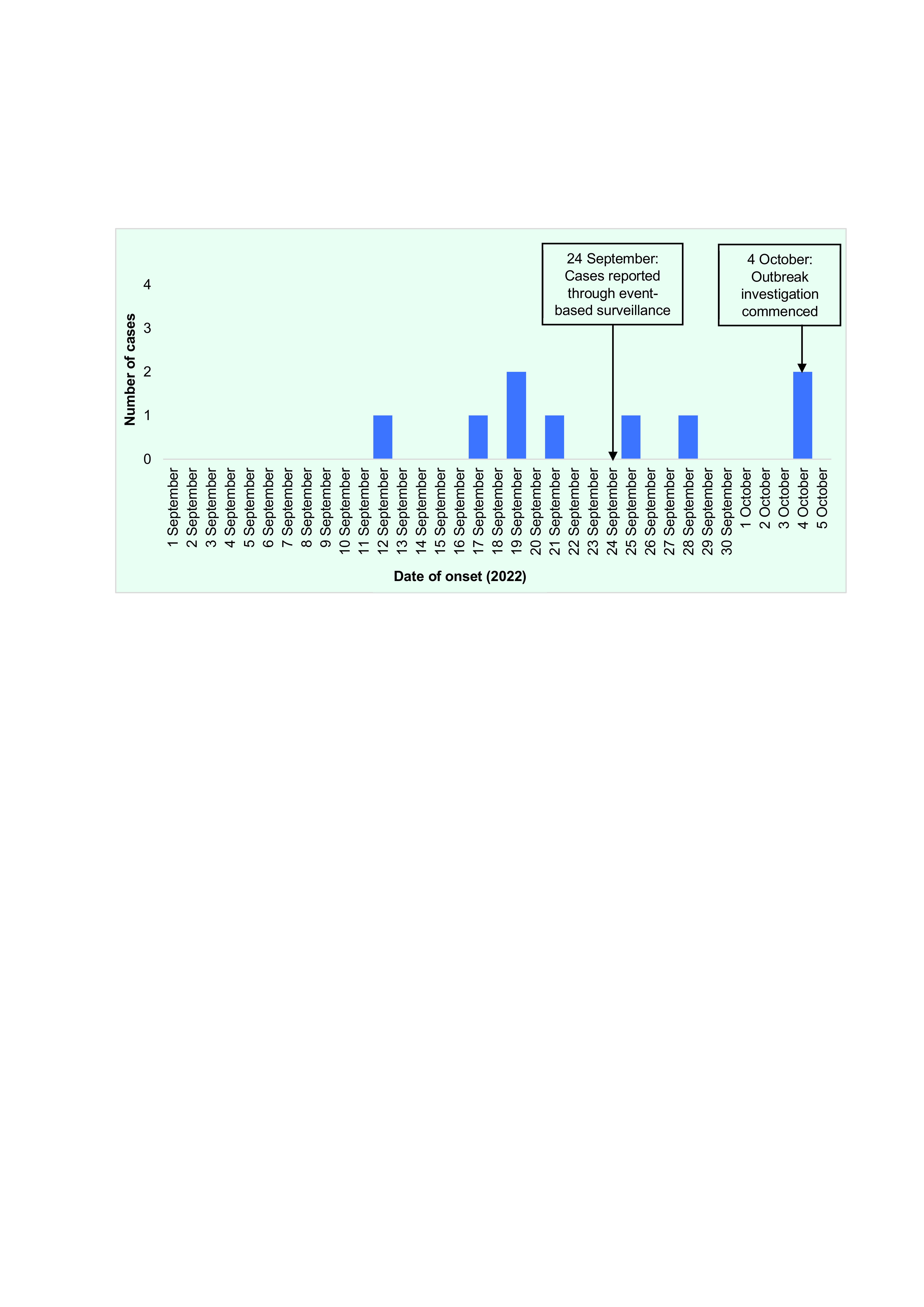
Epidemiological curve of cases of hand, foot and mouth disease (N = 9) by date of onset at a school in Balungao, Pangasinan Province, the Philippines, 1 September-5 October 2022

Nine HFMD cases were recorded at the investigated school during 1 September–5 October 2022. The number of cases peaked during 16–20 September 2022 (**Fig. 1**). The first case had a rash on their hands and feet on 12 September 2022. During the epidemiological investigation, two additional cases manifested signs and symptoms of rash on their hands and feet, as well as having fever and mouth ulcers. It was reported that they had contact with a confirmed HFMD case who is their relative.

The highest number of cases occurred among students in grade 1 (6, 67%), with the majority of cases (7, 78%) occurring in students who were aged 6 years, and five cases (56%) in males. Aside from maculopapular and papulovesicular rashes and mouth ulcers, some cases also developed fever (5, 56%). Rash manifested predominantly on the palms (9, 100%) and fingers (7, 78%). Seven (78%) of the cases reported exposure to a confirmed case of HFMD (**Table 1**). There were no suspected cases among school staff. Surveillance data showed that no HFMD cases were reported in the municipality of Balungao during 2021.

[**insert** Figure 1]

**Table 1:** Characteristics of cases of hand, foot and mouth disease (*N* = 9) at a school in Balungao, Pangasinan Province, the Philippines, 1 September–5 October 2022

Characteristics	No. (%) of cases
Sex	
Male	5 (56)
Female	4 (44)
Age (years)	
6	7 (78)
8	2 (22)
Grade level	
Kindergarten	1 (11)
Grade 1	2 (22)
Grade 3	6 (67)
Reported exposure to a case	7 (78)
Signs and symptoms**^a^**	
Rash	9 (100)
Mouth ulcers	9 (100)
Fever	5 (56)
Loss of appetite	1 (11)
Site of rash**^a^**	
Palms	9 (100)
Fingers	7 (78)
Soles of feet	1 (11)
Buttocks	1 (11)

**^a^** Multiple responses were allowed.

### Key informant interviews

The mother of the index case reported having no known exposure prior to the onset of illness. His teacher noticed he had a cough from 5 to 9 September, but assumed it was just an allergic cough. On 12 September 2022, he developed a papulovesicular rash on his hand; his mother took him to the local medical clinic where he was diagnosed with HFMD.

According to the Municipal Health Officer, no outbreaks of HFMD had been reported in the municipality. The reported cluster of cases at the investigated school was the municipality’s first recorded HFMD event in a school.

The principal, school administrators and teachers reported being aware of a number of students with HFMD at the school during the outbreak, but noted that these were the school’s first reported cases of HFMD. After the first case, teachers began monitoring students for signs and symptoms. At the same time, hybrid learning was implemented in the classroom for grade 1 students because case clustering was identified. Teachers observed that the students did not always wash their hands properly before and after eating.

### Environmental survey

Cases occurred in three grades: kindergarten, grade 1 and grade 3. The school has two washing areas: a common bathroom for each grade level and two common eating areas. The washing area was two to three classrooms away from the classrooms with reported cases of HFMD. The bathroom for grade 1 students was not functional, and the other bathroom was not being properly cleaned.

### Laboratory results

Throat swabs were collected from each of the nine cases. Six (67%) tested positive for CV-A16, two (22%) tested positive for enterovirus and one (11%) was negative for enterovirus RNA.

## DISCUSSION

This outbreak of HFMD in a school in Pangasinan Province, the Philippines, had two causative agents: CV-A16 and enterovirus. Similar studies in a day-care centre in Sydney, Australia, and in Viet Nam also identified CV-A16. (*4*, *5*) The signs and symptoms of the cases were similar to those in other HFMD outbreaks. The mild signs and symptoms reported by the cases concur with the mild and self-limiting signs and symptoms of coxsackievirus infection compared with infection with other types of enterovirus. (*6*) A records review showed that no cases of HFMD had been previously reported in the school or the municipality.

Direct contact with a confirmed case was the source of transmission, and a lack of physical distancing in the classrooms may have contributed to transmission. A study conducted in Beijing, China, found that being in close proximity to someone exhibiting signs and symptoms of HFMD plays a significant role in disease transmission. (*7*) HFMD is spread from person to person by direct contact with the infectious viruses that cause this disease. These viruses are found in nose and throat secretions (i.e. in saliva, sputum and nasal mucus), blister fluid and stool of infected persons. (*8*)

The unknown exposure of the index case may be due to asymptomatic transmission. A study in Bangkok, Thailand, discovered that HFMD can be transmitted by exposure to asymptomatic individuals. (*9*) Although further evidence is needed, the presence of asymptomatic transmission may indicate that this municipality is already prone to HFMD epidemics.

Poor hand-washing practices and minimal disinfection of commonly touched surfaces at the school may have played roles in transmission. The viruses can be spread when infected persons touch objects and surfaces that are then touched by others. (*8*) Ruan et al. discovered that hand-washing by caregivers and children attending preschool significantly reduced the risk of HFMD in the community. (*10*)

This study is only descriptive and is limited in its ability to test a hypothesis and determine risk factors. Despite these limitations, the study was able to identify the pathogen and source of this outbreak. While no additional cases were reported at this school after the outbreak, new cases were recorded at another elementary school and a day-care centre and in one village, suggesting further community spread. We recommended that the local government of Balungao, Pangasinan, engages in health-promotion activities, that schools encourage self-isolation at the onset of symptoms, and that hand-washing facilities are functional and accessible.
